# Acidocalcisome localization of membrane transporters and enzymes in *Trypanosoma brucei*

**DOI:** 10.1128/spectrum.01128-24

**Published:** 2024-10-09

**Authors:** Guozhong Huang, Roberto Docampo

**Affiliations:** 1 Center for Tropical and Emerging Global Diseases, University of Georgia, Athens, Georgia; 2 Department of Cellular Biology, University of Georgia, Athens, Georgia; George Washington University, Washington, DC, USA

**Keywords:** *Trypanosoma brucei*, acidocalcisome, metal transporters, palmitoyl acyltransferase

## Abstract

Acidocalcisomes of *Trypanosoma brucei* are membrane-bounded organelles characterized by their acidity and high content of polyphosphate and cations, like calcium and magnesium. They have important roles in cation and phosphorus storage, osmoregulation, autophagy initiation, calcium signaling, and virulence. Acidocalcisomes of *T. brucei* possess several membrane transporters, pumps, and channels, some of which were identified by proteomic and immunofluorescence analyses and validated as acidocalcisome proteins by their colocalization with the acidocalcisome marker vacuolar proton pyrophosphatase (VP1). Here, we report that a set of membrane transporters and enzymes, which were proposed to be present in acidocalcisomes by the morphological appearance of tagged proteins, colocalize with VP1, validating their character as acidocalcisome proteins.

**IMPORTANCE:**

Acidocalcisomes are acidic organelles rich in polyphosphate and calcium present in a variety of eukaryotes and important for osmoregulation and calcium signaling. Several proteins were postulated to localize to acidocalcisomes based on their morphological characteristics. We provide validation of the localization of ten10 acidocalcisome proteins by their co-localization with enzymatic markers. These findings reveal the roles of acidocalcisomes in the storage of toxic metals, and the presence of enzymes involved in palmitoylation and polyphosphate metabolism.

## INTRODUCTION

Acidocalcisomes are acidic organelles rich in polyphosphate (polyP) and cations, first identified in *T.Trypanosoma brucei* (1), and present in a variety of eukaryotes (2, 3). Their acidification in *T. brucei* is through two electrogenic proton pumps, the multisubunit vacuolar proton ATPase (TbV-ATPase) (1) and the vacuolar proton pyrophosphatase (TbVP1) (4, 5). Biochemical (1) and proteomic (6) studies of acidocalcisomes of *T. brucei,* which is an excellent model organism for their study*,* have led to the discovery of other pumps [Ca^2+^-ATPase (TbPMC1)] (1, 7), channels [potassium channel (TbIRK) (8), inositol 1,4,5-trisphosphate receptor (TbIP_3_R) (9, 10)], membrane transporters [vacuolar iron transporter (TbVIT1) (6), Zn^2+^ transporter (TbZnT1) (6), polyamine transporter (TbPOT) (6), phosphate transporter (TbPho91) (6, 11)], and enzymes involved in polyP synthesis [vacuolar transporter chaperone complex (TbVTC) (11–13)], and degradation [vacuolar soluble pyrophosphatase (TbVSP) (14–16), acid phosphatase (TbAP) (6)].

A recent project aiming at identifying the subcellular localization of all *T. brucei* proteins proposed the localization of a set of proteins to acidocalcisomes based on their morphology and distribution as “multiple point-like or circular foci that cluster away from the nucleus and flagellar pocket” (17, 18). Here, we have investigated the colocalization of these proteins with the acidocalcisome marker TbVP1, a pump that has been reported to localize only to acidocalcisomes of *T. brucei* (4, 5), validated the acidocalcisome localization of 10 of them (Table 1), and discussed the significance of these findings.

**TABLE 1:** Acidocalcisome localization of *T. brucei* proteins, validated by marker (TbVP1) colocalization^*a*^

TriTrypDB gene ID	Annotation (Protein name)	MW (kDa)	TMD
Tb927.11.1260	Copper-transporting ATPase (TbATP7)	102	8
Tb927.8.7460	Zinc transporter 2 (TbZnT2)	51	6
Tb927.5.1260	Sulfate transporter (TbSULT)	63	12
Tb927.9.5490	Magnesium transporter (TbMgT)	76	2
Tb927.11.16630	Major facilitator superfamily protein 1 (TbMFS1)	75	14
Tb927.11.12270	Major facilitator superfamily protein 2 (TbMFS2)	53	10
Tb927.10.12400	Multidrug and toxic extrusion protein (TbMATE)	51	12
Tb927.10.10800	Palmitoyl acyltransferase 2 (TbPAT2)	50	4
Tb927.6.4630	Kinetoplastid-specific phospho-protein phosphatase (TbPpn2)	40	1
Tb927.10.6180	FLA1-like protein (TbFLA1-like)	55	1

^*a*^ TbVP1, *T. brucei* vacuolar proton pyrophosphatase; MW, molecular weight; TMD, transmembrane domains.

## RESULTS

The newly proposed acidocalcisome proteins (18) include one pump (copper-transporting ATPase-like protein, TbATP7, Tb927.11.1260), six membrane transporters (zinc transporter 2, TbZnT2, Tb927.8.7460), sulfate transporter (TbSULT, Tb927.5.1260), magnesium transporter (TbMgT, Tb927.9.5490), major facilitator superfamily protein (TbMFS1, Tb927.11.16630), drug resistance protein (TbMFS2, Tb927.11.12270), and membrane transporter protein (TbMATE, Tb927.10.12400) (Fig. S1), four enzymes (palmitoyl acyltransferase 2 (TbPAT2, Tb927.10.10800), kinetoplastid-specific phospho-protein phosphatase (TbPpn2, Tb927.6.4630), oxidoreductase (TbOR, Tb927.10.9560), and serine-threonine kinase receptor-associated protein (TbSTRAP, Tb927.11.1890), one FLA1-like protein (TbFLA1-like, Tb927.10.6180) (Fig. S1), and six hypothetical proteins (Tb927.4.860, Tb927.4.5335, Tb927.8.6990, Tb927.9.12070, Tb927.10.11810, and Tb927.11.8250). Of these proteins, TbPpn2, TbPAT2, and a FLA1-like protein were also reported in proteomic studies of acidocalcisomes of *T. brucei* (6). We therefore investigated the colocalization of the annotated proteins with TbVP1 to validate their character as acidocalcisome proteins.

### Copper-transporting ATPase

**FIG 1:**
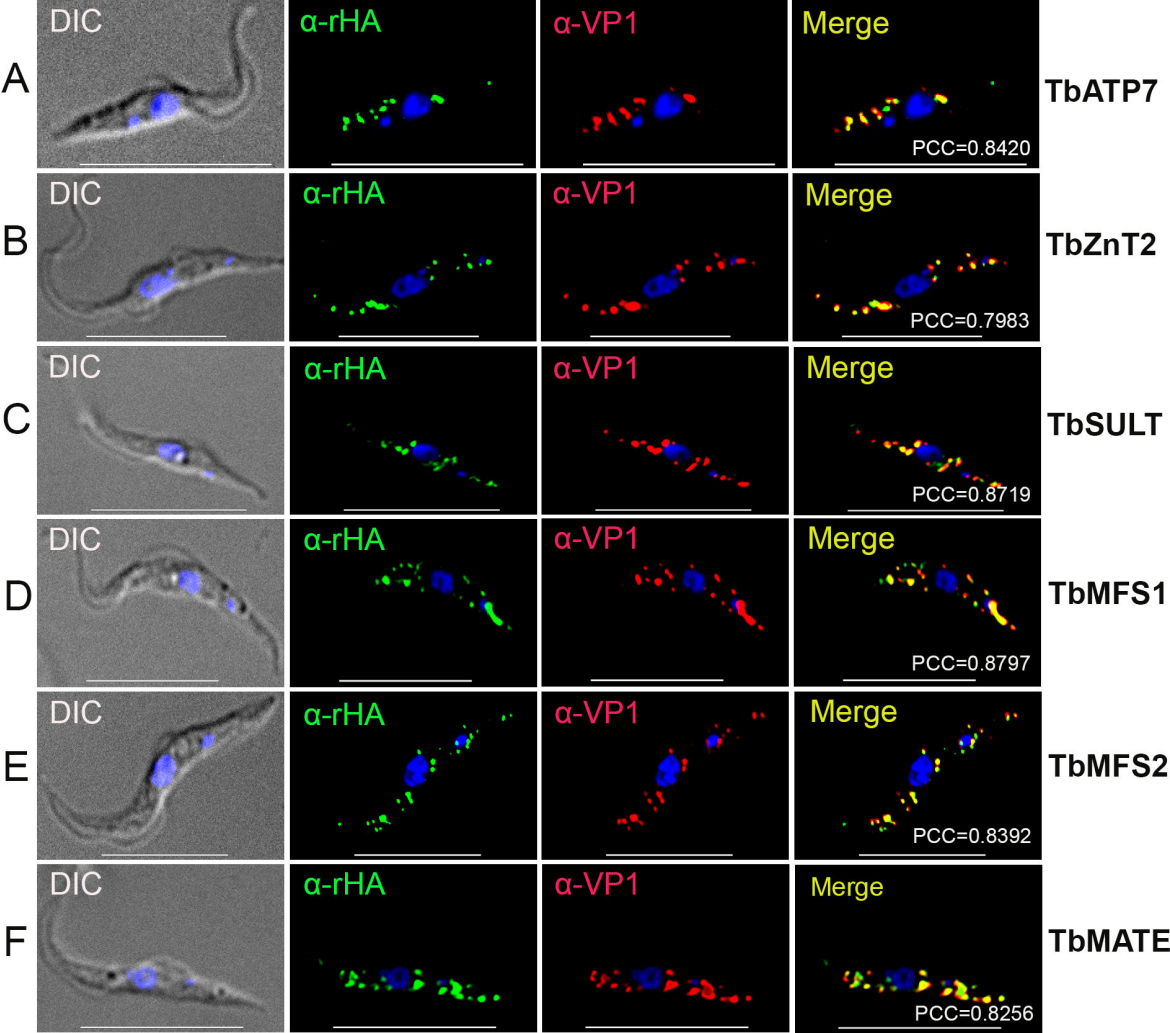
Immunofluorescence microscopy of six membrane transporters. TbATP7 (**A**), TbZnT2 (**B**), TbSULT (**C**), TbMFS1 (**D**), TbMFS2 (**E**), and TbMATE (**F**) were C-terminally smHA-tagged *in situ* and colocalized with TbVP1 in acidocalcisomes of procyclic form (PCF) trypanosomes (Pearson’s correlation coefficients of 0.8420, 0.7983, 0.8719, 0.8797, 0.8392, and 0.8256, respectively). α-rHA, rabbit anti-HA antibody. Yellow in merge images indicates colocalization. Scale bars for (A–F)  =  10 µm.

Copper (Cu) is essential for most cells, and orthologs to the mammalian Cu ATPases, ATP7A and ATP7B, were previously reported in several trypanosomatids (19, 20). They were localized to vacuoles, presumably acidocalcisomes (19), and the plasma membrane (19, 20), and possess eight transmembrane domains (TMDs) (Fig. S1). Excess copper ions are toxic to the cells, and copper ATPases can sequester them in vacuoles, preventing Fenton reactions and oxidative stress (21). To investigate the localization of TbATP7, its C-terminus was tagged in procyclic form (PCF) trypomastigotes with a spaghetti monster (sm) hemagglutinin (HA) tag (22) using homologous recombination with the endogenous locus. Western blot analysis confirmed the expression of the protein of the expected size (Fig. S2A). TbATP7 localized to acidocalcisomes, as demonstrated by colocalization of rabbit (Fig. 1A) or mouse (Fig. S3A) antibodies against HA with antibodies against *T. brucei* acidocalcisome marker TbVP1.

### Membrane transporters

**FIG 2:**
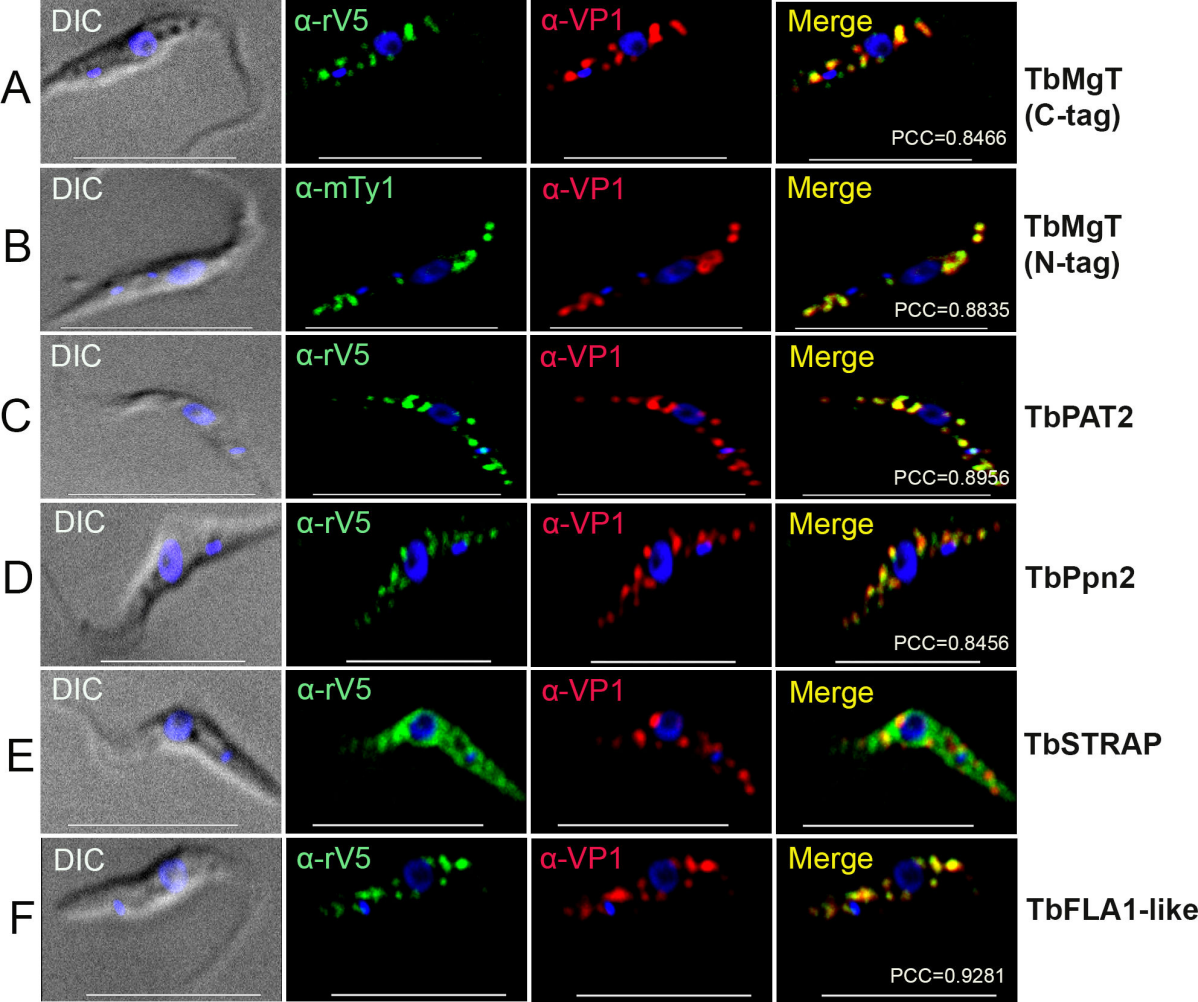
Immunofluorescence microscopy of two membrane proteins and three enzymes. TbMgT C-tag (**A**), TbMgT N-tag (**B**), TbPAT2 (**C**), TbPpn2 (**D**), TbSTRAP (**E**), and TbFLA1-like (**F**) were C-terminally smV5-tagged (**A, C–F**) or N-terminally Ty1-tagged (**B**) *in situ* and colocalized with TbVP1 in acidocalcisomes of procyclic form (PCF) trypanosomes [Pearson’s correlation coefficients (PCCs) were 0.8466 (TbMgT C-tag), 0.8835 (TbMgT N-tag), 0.8956 (TbPAT2), 0.8456 (TbPpn2), and 0.9281 (TbFLA1-like)]. α-rV5, rabbit anti-V5 antibody; α-mTy1, mouse monoclonal anti-Ty1 antibody. TbSTRAP has cytoplasmic localization. Yellow in merge images indicates colocalization. Scale bars for (A–F)  =  10 µm.

Using a similar approach to that used with TbATP7, we endogenously tagged the C-terminus of TbZnT2 of PCF, a protein of 6 TMD (Fig. S1) that shares 86% identity and 90% similarity to the previously reported zinc transporter TbZnT1 (6) (Fig. S4A), with the smHA tag and colocalized it with TbVP1 (Fig. 1B; Fig. S3B). Western blot analysis showed a band of the expected size (Fig. S2A). Similar results were obtained after endogenously tagging TbSULT (Fig. 1C; Fig. S3C), TbMFS1 (Fig. 1D; Fig. S3D), TMFS2 (Fig. 1E; Fig. S3E), and TbMATE (Fig. 1F; Fig. S3F), which all colocalized with TbVP1. For localization studies of TbMgT, we endogenously tagged the C-terminus or N-terminus of the protein with smV5 or Ty1 tags, respectively, and colocalized it with TbVP1 (Fig. 2A and B). Western blot analyses of all these proteins revealed bands of the expected size (Fig. S2B through G).

### Enzymes

*T. brucei* has 12 palmitoyl acyltransferases (TbPAT1-12) (23), and previous studies in *Trypanosoma cruzi* localized the ortholog of TbPAT2 to a cytoplasmic organelle compatible with acidocalcisomes (24). Figure 2C and Fig. S5A show the colocalization of smV5- (Fig. 2C; Fig. S5A)-tagged TbPAT2 with TbVP1, and Fig. S2H shows a control western blot analysis.

Tb927.6.4630 is annotated in TriTrypDB as kinetoplastid-specific phospho-protein phosphatase and appears to have homology to the *Saccharomyces cerevisiae* endopolyphosphatase 2 (ScPpn2) (Fig. S4B), which localizes to its acidocalcisome-like vacuole (25), and we tentatively named it TbPpn2. Figure 2D shows its colocalization with TbVP1 in *T. brucei*, and a western blot analysis is shown in Fig. S2I.

We were unable to obtain the N- or C-terminus tagging of the oxidoreductase (TbOR, Tb927.10.9560) while tagging of the serine-threonine kinase receptor-associated protein (TbSTRAP, Tb927.11.1890) resulted in predominant cytosolic labeling, with punctate-like pattern probably due to deconvolution (Fig. 2E). A western blot analysis is shown in Fig. S2J, and although a band of the expected size is shown, other proteins are also labeled.

### FLA1-like protein

FLA1-like (Tb927.10.6180) is a transmembrane protein that was found to have NHL-repeats as the FLA1 protein, which is important for flagellum attachment and cell division (26). It also colocalizes with TbVP1 to acidocalcisomes (Fig. 2F), and a western blot analysis shows a band of the expected size (Fig. S2J).

## DISCUSSION

Of the 12 proteins investigated in this work, we found evidence of acidocalcisome colocalization with TbVP1 in 10 of them (Table 1). We were not able to confirm the colocalization of TbOR with TbVP1, while TbSTRAP showed predominant cytosolic localization and no evident colocalization with TbVP1. The results in general support the identification of acidocalcisomes of *T. brucei* based on their morphological appearance.

The identification of several metal transporters in acidocalcisomes [copper ATPase TbATP7; Zn^2+^ transporters (TbZbT1 (6), TbZnT2), vacuolar iron transporter, TbVIT (6)] supports their role in sequestration of toxic metals. For example, iron (Fe^3+^) and copper (Cu^2+^) can be reduced by superoxide anion radical (O_2_.^-^), mainly generated as a by-product of mitochondrial respiration, according to this reaction:


$${Fe}^{3+}/{Cu}^{2+}+O_{2}^{-}⟶{Fe}^{2+}/{Cu}^{+}+O_{2}$$

These reduced metals are then able to decompose hydrogen peroxide with the generation of hydroxyl radical and hydroxyl anion (Fenton reaction):


$${Fe}^{2+}/{Cu}^{+}+H_{2}O_{2}⟶{Fe}^{3+}/{Cu}^{2+}+{HO}^{\cdot }+{HO}^{-}$$

Superoxide anion radical is not very reactive except when combined with nitric oxide (NO^.^) to form peroxynitrite or with iron-sulfur clusters in proteins, and it generates hydrogen peroxide by a reaction catalyzed by the superoxide dismutase. Hydrogen peroxide is poorly reactive but is cell permeable. In contrast, hydroxyl radical is extremely reactive and can oxidize lipids, proteins, and nucleic acids (27).

Zn^2+^ is a catalytic/structural cofactor of many metalloproteins, has also a regulatory role, and may inhibit metabolic enzymes when at high concentrations (28). To prevent the accumulation of free metal ions to toxic levels, metazoan cells bind them to scavenger proteins such as metallothioneins [for Zn^2+^ and Cu^2+^ (29, 30)] and ferritin [for Fe^3+^ (31)]. However, there are no gene homologs encoding these scavengers in trypanosomatids, and sequestration in acidocalcisomes and binding to polyP could be their only mechanism of detoxification and prevention of oxidative stress.

Mg^2+^ is a cofactor in many enzymatic reactions. In fungal and plant cells, most of Mg^2+^ is sequestered in the vacuole buffering variations in environmental Mg^2+^ (32). When environmental Mg^2+^ is abundant, it is sequestered in the vacuole to prevent toxicity caused by imbalance among several minerals; and when environmental Mg^2+^ is deficient, vacuolar Mg^2+^ is mobilized (32). A similar situation would occur in trypanosomatids where Mg^2+^ is also highly concentrated within acidocalcisomes (4) mainly bound to polyP (33), and TbMgT could be responsible for its uptake.

Sequestration of sulfur, in the form of sulfate, by the vacuoles of fungi and plants (34), as well as by acidocalcisomes of trypanosomatids through TbSULT, could potentially serve as a buffering mechanism of this important element.

Major facilitator superfamily (MFS) proteins are responsible for transporting a broad range of substrates. This includes a variety of sugars, drugs, and other hydrophobic substrates, inorganic or organic anions or cations, peptides, nucleosides, and aromatic acids (35). For example, TbMFS1 and TbMFS2 could be involved in the influx or efflux of acidocalcisome components such as inorganic and organic cations or anions, phosphate, and polyphosphatespolyP, for which there are no specific transporters.

Multidrug and toxic compound extrusion (MATE) proteins are also part of a large family of secondary active transporters present in the three domains of life (36). Some are important for transporting cationic compounds or for the accumulation of metabolites in organelles such as the vacuole of plants (36), and TbMATE could also have such a role in acidocalcisomes.

One interesting property of protein palmitoylation is the localization of some palmitoyl acyltransferases (PATs) in the same subcellular compartment as their substrates. Several acidocalcisome proteins from *T. brucei* have been reported to be palmitoylated [TbATP7, TbVTC1, TbVTC4, TbSULT, TbPpn2 (23)], and TbPAT2 was shown to localize to acidocalcisomes.

TbPpn2 has similarity to the yeast Zn^2+^-dependent endopolyphosphatase Ppn2 (Fig. S3B), which localizes to the yeast acidocalcisome-like vacuole and could be involved, as the yeast enzyme, in mobilizing polyP in response to phosphate scarcity (25).

We did not find evidence of the colocalization of the *T. brucei* serine-threonine kinase-associated receptor (TbSTRAP) with TbVP1. In mammalian cells, STRAP localizes to both the cytoplasm and nucleus and was originally identified as a transforming growth factor β-interacting protein (37). It also interacts with other proteins and plays roles in cell proliferation and apoptosis (37).

Finally, the FLA-like protein colocalized with TbVP1 but its function is unknown.

In conclusion, our results validate the presence of 10 new proteins in acidocalcisomes of *T. brucei,* provide evidence of sequestration of several metals in acidocalcisomes, which could prevent their toxicity, and describe new enzymes with active roles in acidocalcisome metabolism.

## MATERIALS AND METHODS

### Cell transfection

*T. brucei* wild-type PCF trypanosomes were grown in SDM-79 medium (38), supplemented with hemin (7.5 µg/mL) and 10% heat-inactivated fetal bovine serum at 27°C. Mid-log phase PCF (∼5 × 10^6^ cells/mL) were harvested by centrifugation at 1,000 *× g* for 7 min, washed with Cytomix buffer (2 mM EGTA, 3 mM MgCl_2_, 120 mM KCl, 0.5% glucose, 0.15 mM CaCl_2_, 0.1 mg/mL bovine serum albumin (BSA), 10 mM K_2_HPO_4_/KH_2_PO_4_, 1 mM hypoxanthine, 25 mM HEPES, pH 7.6) and resuspended in 0.45 mL of the same buffer at a cell density of 2.5 × 10^7^ cells/mL. The washed cells were mixed with purified PCR products (10 µg) in a 0.4 cm electroporation cuvette and subjected to two pulses from a Bio-Rad Gene Pulser electroporator set at 1.5 kV and 25 µF. The stable transformants were obtained in SDM-79 medium supplemented with 15% fetal bovine serum (FBS) plus 5 µg/mL puromycin.

### PCR-mediated *in situ* epitope tagging of *T. brucei* genes

The one-step epitope-tagging protocol reported by Oberholzer et al. (39) and Dean et al. (40) was used to produce N- or C-terminal epitope-tagging cassettes for transfection of *T. brucei* PCF trypanosomes. In the N-terminal Ty1-tagging approach, the PCR forward and reverse primers (Table S1) consist of the last 140 nucleotides of the target gene’s 5′ untranslated region (UTR) and the first 140 nucleotides of the target gene’s open reading frame (ORF) without the stop codon in reverse complement, respectively, followed by the 18–20 nucleotides of the backbone sequences of pPOTv6-Puro-Puro-mNG (40). In the C-terminal “Spaghetti monster” fluorescent proteins (smFPs, smHA, and smV5) tagging approach (41), the PCR forward and reverse primers (Table S1) included terminal 100–120 nucleotides of each ORF before its stop codon and the reverse complement of the first 100–120 nucleotides of the 3′ UTR, respectively, followed in frame by the 21–26 nucleotides of the backbone sequences of pMOTag2mH or pMOTag2mV vector (42). The epitope-tagging cassettes containing an antibiotic selection marker (puromycin resistance gene) were generated for cell transfection by PCR using pPOTv6-Puro-Puro-mNG (40), pMOTag2mH, or pMOTag2mV (42) as a template with the corresponding PCR primers of the gene. In this study, the utilization of smV5 or smHA tagging depended on the availability of anti-HA or anti-V5 antibodies for their detection.

### Immunofluorescence microscopy

PCF trypanosomes were washed with phosphate bufferf saline (PBS) and then fixed with 4% paraformaldehyde in PBS at room temperature for 1 h. The fixed parasites were washed twice with PBS, allowed to adhere to poly-l-lysine-coated coverslips, and permeabilized with 0.3% Triton X-100/PBS for 3 min. After blocking with PBS containing 3% BSA, 1% fish gelatin, 50 mM NH_4_Cl, and 5% goat serum for 1 h, trypanosomes were stained in 1% BSA/PBS with the Guinea pig polyclonal antibody against TbVP1 (1:300), rabbit anti-HA antibody (1:1,000), purified HA.11 clone 16B12 mouse monoclonal antibody against HA (1:50), rabbit anti-V5 antibody (1:500), mouse monoclonal anti-V5 antibody (1:100), and mouse monoclonal anti-Ty1 antibody BB2 (1:100) for 1 h. After thoroughly washing with PBS containing 1% BSA, cells were incubated with Alexa 488-conjugated goat anti-mouse or anti-rabbit antibody, and Alexa 546-conjugated goat anti-Guinea pig at 1:1,000 for 1 h. The cells were counterstained with 4',6-diamidino-2-phenylindole (DAPI) before mounting with Gold ProLong Gold antifade reagent (Molecular Probes). Differential interference contrast and fluorescent optical images were captured using an Olympus IX-71 inverted fluorescence microscope with a Photometrix CoolSnap^HQ^ CCD camera driven by DeltaVision software (Applied Precision, Seattle, WA). Images were deconvolved for 15 cycles using Softwarx deconvolution software. Pearson’s correlation coefficients were calculated using the Softwarx software by measuring the images of whole cells or specific cell-staining regions. More than 20 cells were randomly observed and analyzed.

### Western blot analyses

PCF trypanosomes were harvested and washed twice in PBS. The washed cells were lysed with RIPA buffer (150 mM NaCl, 20 mM Tris/HCl, pH 7.5, 1 mM EDTA, 1% SDS, and 0.1% Triton X-100) containing protease inhibitor tablet (Roche) in ice for 1 h. The protein concentration was determined using Pierce BCA protein assay kit with the microplate reader. The total cell lysates were mixed with 2× Laemmli sample buffer (Bio-Rad) at a 1:1 ratio (volume/volume) and directly loaded. The separated proteins were transferred onto nitrocellulose membranes using a Bio-Rad transblot apparatus. The membranes were blocked with 10% non-fat milk in PBS containing 0.5% Tween-20 (PBS-T) at 4°C overnight. The blots were incubated with purified HA.11 clone 16B12 mouse monoclonal antibody against HA (1:1,000), mouse monoclonal anti-V5 antibody (1:2,500), mouse monoclonal anti-Ty1 antibody BB2 (1:1,000), or mouse antibodies against tubulin (1:10,000) for 1 h. After five washings with PBS-T, the blots were incubated with horseradish peroxidase-conjugated anti-mouse IgG (H + L) antibody at a dilution of 1:15,000 for 1 h. After washing five times with PBS-T, the immunoblots were visualized using Pierce ECL western blotting substrate according to the manufacturer’s instructions.

## SUPPLEMENTAL MATERIAL

Supplemental material spectrum.01128-24-s0001.pdf Fig S1 to S5; Table S1.

ASM does not own the copyrights to Supplemental Material that may be linked to, or accessed through, an article. The authors have granted ASM a non-exclusive, world-wide license to publish the Supplemental Material files. Please contact the corresponding author directly for reuse.
