# Supplementary material for: Acidocalcisome localization of membrane transporters and enzymes in Trypanosoma brucei
Source: Microbiol Spectr. 2024 Oct 9;12(11):e01128-24. doi: 10.1128/spectrum.01128-24 (PMC11537032; doi:10.1128/spectrum.01128-24)
Supplement: Supplemental material — Fig S1 to S5; Table S1. [file spectrum.01128-24-s0001.pdf]

Acidocalcisome localization of membrane transporters and enzymes in *Trypanosoma brucei*

Guozhong Huang[1] and Roberto Docampo[1,2]*

[1]Center for Tropical and Emerging Global Diseases and [2]Department of Cellular Biology,

University of Georgia, Athens, Georgia, 30602

Table S1. Primers used in this study

Name	Tagging	Primer sequence (5' to 3')
TbATP7-TF	C-terminal	CCCCTGTCGTCGCCGGGGTTGCAATGATTCTTTCGAGCCTTAGTGTGCTTATGTCTAGCCTTTCCATTCGCTGTTTCTCACCTTACAAACGGGAACAGTTCATTGATATCATTGGTACCGGGCCCCCCCTCGAG
TbATP7-TR	C-terminal	AAAACTATCATACTCCTTGTCTCCTTCTCCTCACCCGTTATTATTACCCTTGTTATTACCATTATTTTTCATCACAATTATTAGTGCCGTTGTTGAGTATTCTTTCACCAATTTGGCGGCCGCTCTAGAACTAGTGGAT
TbZnT2-TF	C-terminal	CACAGGAGGTTTGCAAGAGATTTGGCATCACCCATACGACCATTCAGGTGGACACGGTGGAAAATGGGGCAGGTCTATGTCACTCCCTGTGCGCCTCGGCACAAACTATCGCCGGTACCGGGCCCCCCCTCGAG
TbZnT2-TR	C-terminal	CATCTGCTCACTTACTGTGGAAGGATAGAAAGAAAAATCAGTGGGCCGAATGTGTGTGTGTGCCCTTGTACACATCATACACTTCACCCATCTGGTGTTCCACCATTGCGCAGTGGCGGCCGCTCTAGAACTAGTGGAT
TbSULT-TF	C-terminal	ACCGCGAGAAAAATTGGGTTGTCGATGCTATTAACGCCCAACACCTTCGAAACAGCCTCGCCTTGTGCACACCCTTCCATGATTTGCACAAGTTGGAGTATCAAGAATTGTACGGTACCGGGCCCCCCCTCGAG
TbSULT-TR	C-terminal	GAAAGAAGGAAGGAAGAAGAGAGTGCTGTATACATATACATATATATATATATATATATATATATTATTTATTTATTTATTTATTTCGGATAATCCGTACATAATGCCCATCATGGCGGCCGCTCTAGAACTAGTGGAT
TbMFS1-TF	C-terminal	TACACAAGCAGAGGCATGCGTCTAACGAGGCTCCCGTAAAGGCGGATCACATCGGCTTCAACTTCAAAAGAAGTGGAAAACAGAGTCCAGCAGGTGGTTCTGCAGCAAATATTGGTACCGGGCCCCCCCTCGAG
TbMFS1-TR	C-terminal	ATCTTTTTTTTTTCTTCGCCCTGCGGCTGCAGAGGAGAGAGTTAACGAATATGCGGTATGCAGCGGCAAGAAAGAAAAAAAAAAAGAGAAAATAGAGAACAAATAAAGCGCCTGTGGCGGCCGCTCTAGAACTAGTGGAT
TbMFS2-TF	C-terminal	CCGTGTCTCTCATGATTCACCGTTTCGACTTTGCAATTTGCAGCAATTTTTGGGGCGTGTTGGGCATTTTTGCGGCGGTTTGGGGCTGGTACGGAGTCCCAAATCCCACTCGCGGTACCGGGCCCCCCCTCGAG
TbMFS2-TR	C-terminal	CTCTTGACATCATCAGACACTTTCATGCAGGTGTTTCTCTACTAGTACCGATTAAACGCTTTTTTTTTTTTGCGCGTGTGGTGTGCCGCCCTCCTCCACACGCACCCCCTTCTTGGCGGCCGCTCTAGAACTAGTGGAT
TbMATE-TF	C-terminal	TGTTTGAAGTGATTTTGCTTTATTGTCTGATGAGCCGCTGGGATTGGAAGAAACTCGCACAAAAAGCTCTTGAGTTGACAGAAGGTGAGGAAAAGGCCCTGCTTTCCACTAACGGTACCGGGCCCCCCCTCGAG
TbMATE-TR	C-terminal	CACACGCGGTAAGGTTGCTGCTTACCGTAAGTTGAATTTTATTCCTCTTCCATTTATCAAGCTACACCTTGACATGTGAAGTTGTTCACATCGCCGTGTACGTGGCCACGACGTGGCGGCCGCTCTAGAACTAGTGGAT
TbMgT-CTF	C-terminal	TCTTTCCTTGCCGCAGTGTATGGTATGAATTTTCCCCACATACCTGAGTTGCAATGGGCATGGGGCTATCCATGCTATTGGTTGGCATGTATTGTGGCTTGTGTCCTCATATATCTTTACATGTACCGACGAGGACTTCTGGAGGGTACCGGGCCCCCCCTCGAG
TbMgT-CTR	C-terminal	CCCAAAAGTAACAAAAAAAAATCATAAACGGTAAAAGAGTGAAAGAAGAAATGAGGTATCGCCTCCTTCAAGTTGGATCTTCTTTTTTTTTTCTTTCTTTCTTTTCTTTTGGTGTTGACGCGGGCAACAGAATCATGGTATTGGCGGCCGCTCTAGAACTAGTGGAT

TbMgT-NTF	N-terminal	TAGGAGGGAAGAAAAGGGGTTTGAATTGGCGCTGCAACGGCGTAGTGACTCGTAGCGCTGCACGACCACTTGTAGCTTGCAAGCTACACTTTTACTTATTATTATTATTATTAAATACGCTCCTTTTGCCGCCTAAGGTACTAAGTATAATGCAGACCTGCTGC
TbMgT-NTR	N-terminal	TTTATGAAGCTCATACAAGACTTTGATACTCTGAACCACTTGCTCTGCGTGGCGGGTACTCATTCCTGTTGCCGCCGGTGAATCCAACAGTGTCGCCGATCCCGGTGACGCTTCACGCGGCCCCTGCTCCACGTTCAACGATCCACTACTACCCGATCCTGATCC
TbPAT2-TF	C-terminal	AGGGGCGTGGGGTGTGTAATGTTTACCACCTGTCACAGGTTTCGAGTCATGTTGGAAGTCTATTAGGTCAAACATTTCCAACTGCGGTACCGTTGACAAGTCCTACTCCTGTTGGTACCGGGCCCCCCCTCGAG
TbPAT2-TR	C-terminal	GAGGGGACAGAATGAAACATAAAAGGCAAAAGTCTAGCTTCCTGCATCCCTCTGCACGCGAATCAAAGGATCTCCCAAACTATCTCCACCGTGTGCTAAGTGCTAAACGCCCATGGCGGCCGCTCTAGAACTAGTGGAT
TbPpn2-TF	C-terminal	TTGTCATCATTCTCGTTTCTTTGGTATTTATTGGGAAGTGGGCAGTATCGTTTATCTGCGGTGGTAACGGCGGCACTCCCGGTAATCGACGATATGGGACTTTTTCCCCTGTGGGTACCGGGCCCCCCCTCGAG
TbPpn2-TR	C-terminal	CGCGAAAAAGAAAAACACGCAGCACATGCACATTAAACACAGCACATTGACCTCTTCTGCATGCGGACCCTTGACTTAATCGGCAGCTGACTCCTCCCGTCGATTCTGAGCTCTGGCGGCCGCTCTAGAACTAGTGGAT
TbOR-CTF	C-terminal	GAACACTACAGAGCTGTATTGACCCTTCGAAGTTTAAAGGGTTGGAAGCTGTGGCTGATGCGGTGGAGTATATGTACGAAAGGCGGAATAAGGGAAAGGTCATCGTTGAGATTGGTACCGGGCCCCCCCTCGAG
TbOR-CTR	C-terminal	GAGCCACACGCACAGAGACAATAATAAGAATAATACCAGAGGACCACCATCCATTCACCTTCTCCCTTGTTAGCGCAGGGCTGCATCACAACCAAACGCACATAGAAAGATAATGGCGGCCGCTCTAGAACTAGTGGAT
TbOR-NTF	N-terminal	ACAATCGCCAATGTTCGCGTCCATGTAGGCGTTGGTGTTCTTATCTTCCTTACCGTTTATTTACAAGGAGGAGAAAGCAAAGTCTTTTTTTTAAAAGCTGACAGCCCACTGGCGTATAATGCAGACCTGCTGC
TbOR-NTR	N-terminal	AACCGGTTTTTCACCAGAAGCTCAGTTGACTTAAGATAGGAAGGGATAGGAGATGACAGAACCTCAGTAGCTTCACGGAAGTTTGAGGACAGTCTCTTCGCTACAATTTTCCTCTGACTACCCGATCCTGATCC
TbSTRAP-TF	C-terminal	TTTTTCATATACGCTGGGCACCAGACGGTAAATCATTTACCTCCGGCGCAGAAGATGGTATGGCTCGTATTTGGCCGTCTCACGACATAATCGAGACATACGATAACGAGAACGGTACCGGGCCCCCCCTCGAG
TbSTRAP-TR	C-terminal	CATTTGGGTTTTAAAATTTTTTTTGGTAGTGATCTTCGGGGACAATCAGGTGAGGTGCCTAAATTTGTGGTCGACGGAAAGTTACCAAAAGTGTCCTCTGACTGTTGCTTTGTTGGCGGCCGCTCTAGAACTAGTGGAT
TbFLA1-like-TF	C-terminal	TCCGTCGTCTGAAGAGCAGCTACAAGAACTACTTTAAGCATATGGGAGCACGAGAGGCTGAGGATGTTGAGCTTTTGCAACATGAAATTAACAGTGGATACGGAACGAACAACGGTACCGGGCCCCCCCTCGAG
TbFLA1-like-TR	C-terminal	ACAGGCGCATAGACATGTAAACGAAGTAGGGAACAAAGAATAATGGAAGTGCAGGCACATGGCAGGTAACGGCAATATCAATTCTCCCATGTACTCCTGAGGAAGAAATAAAATGGCGGCCGCTCTAGAACTAGTGGAT

The last letter "F"s or "R"s of the primer names stand for "forward" and "reverse" primers, respectively.

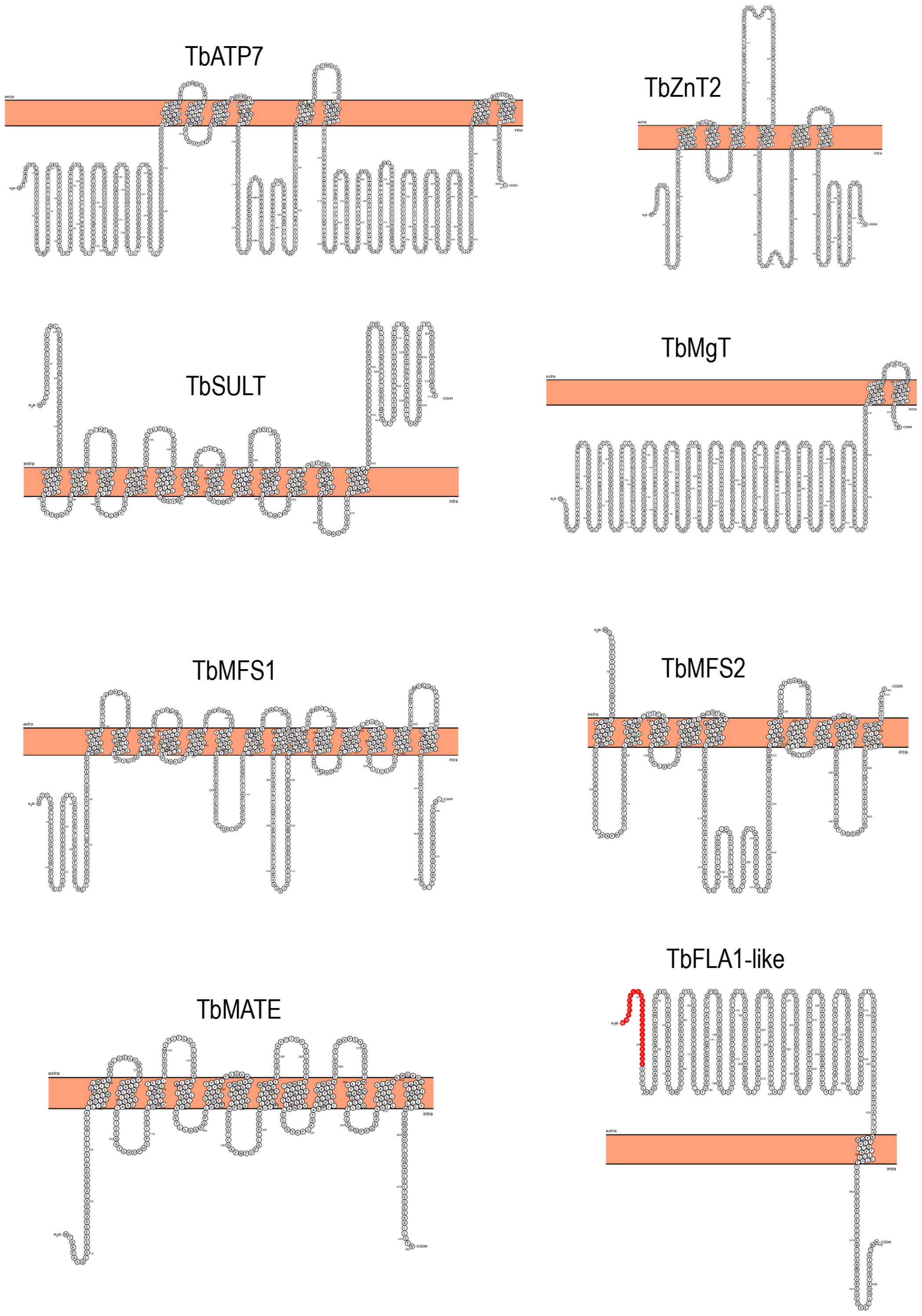

FIG S1 Topology models. Putative topology models of eight membrane proteins predicted with Protter (43). A putative signal peptide sequence is marked in red.

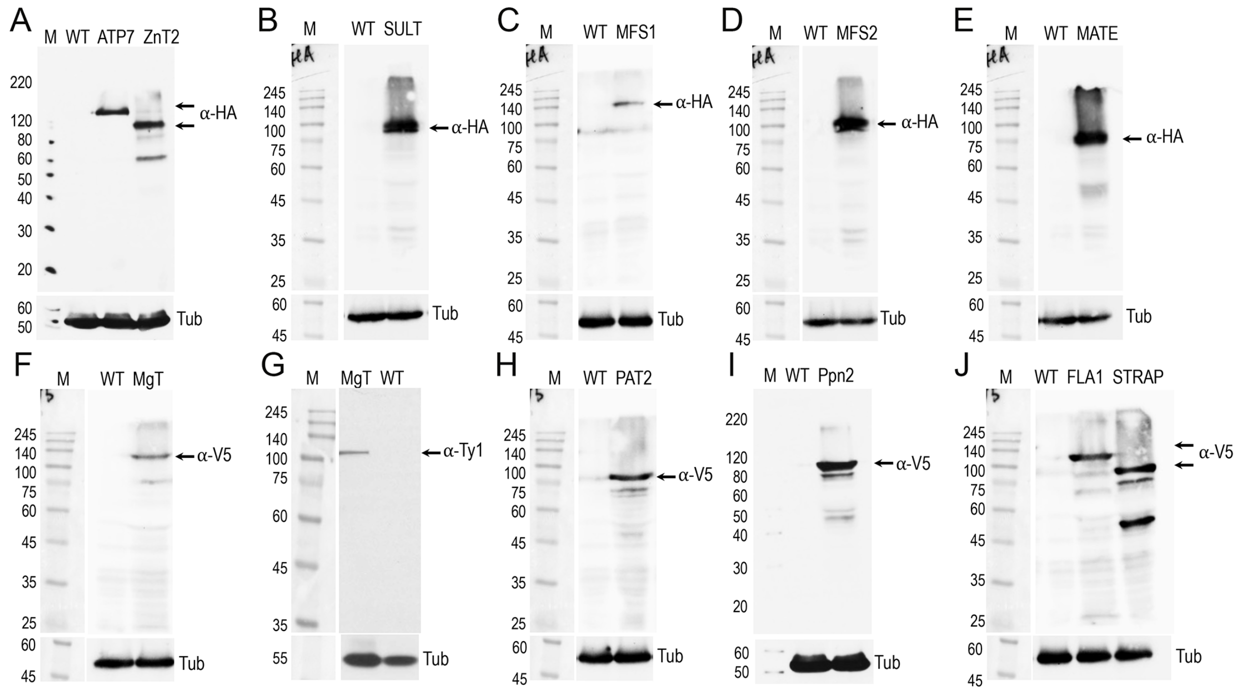

FIG S2 Western blot analyses. Tagging was confirmed by Western blot analyses using monoclonal anti-HA (A-E), anti-Ty1 (G) or anti-V5 (F, H-J) antibodies. MagicMark XP or PureView Prestained Protein ladders are at the left side, arrows showing the corresponding bands. Tubulin (Tub) was used as a loading control (bottom).

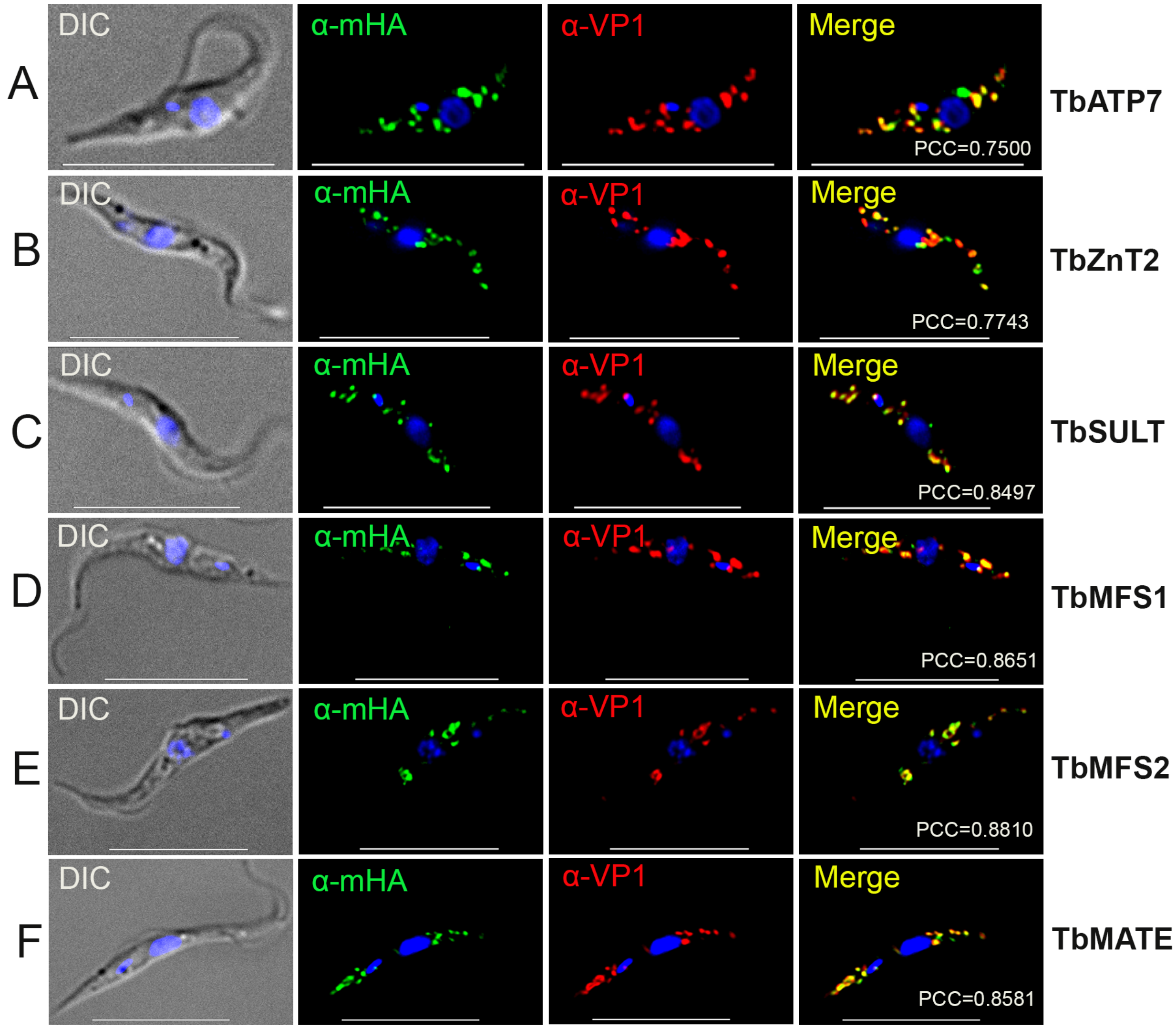

FIG S3 Immunofluorescence microscopy of six membrane transporters. TbATP7 (A), TbZnT2 (B), TbSULT (C), TbMFS1 (D), TbMFS2 (E) and TbMATE (F) were C-terminally smHA-tagged in situ and co-localized with TbVP1 in acidocalcisomes of PCF trypanosomes (Pearson's correlation coefficients of 0.7500, 0.7743, 0.8497, 0.8651, 0.8810 and 0.8581, respectively). α-mHA, mouse monoclonal anti-HA antibody. *Yellow* in merge images indicate co-localization. *Scale bars* for (A–F)=10 µm.

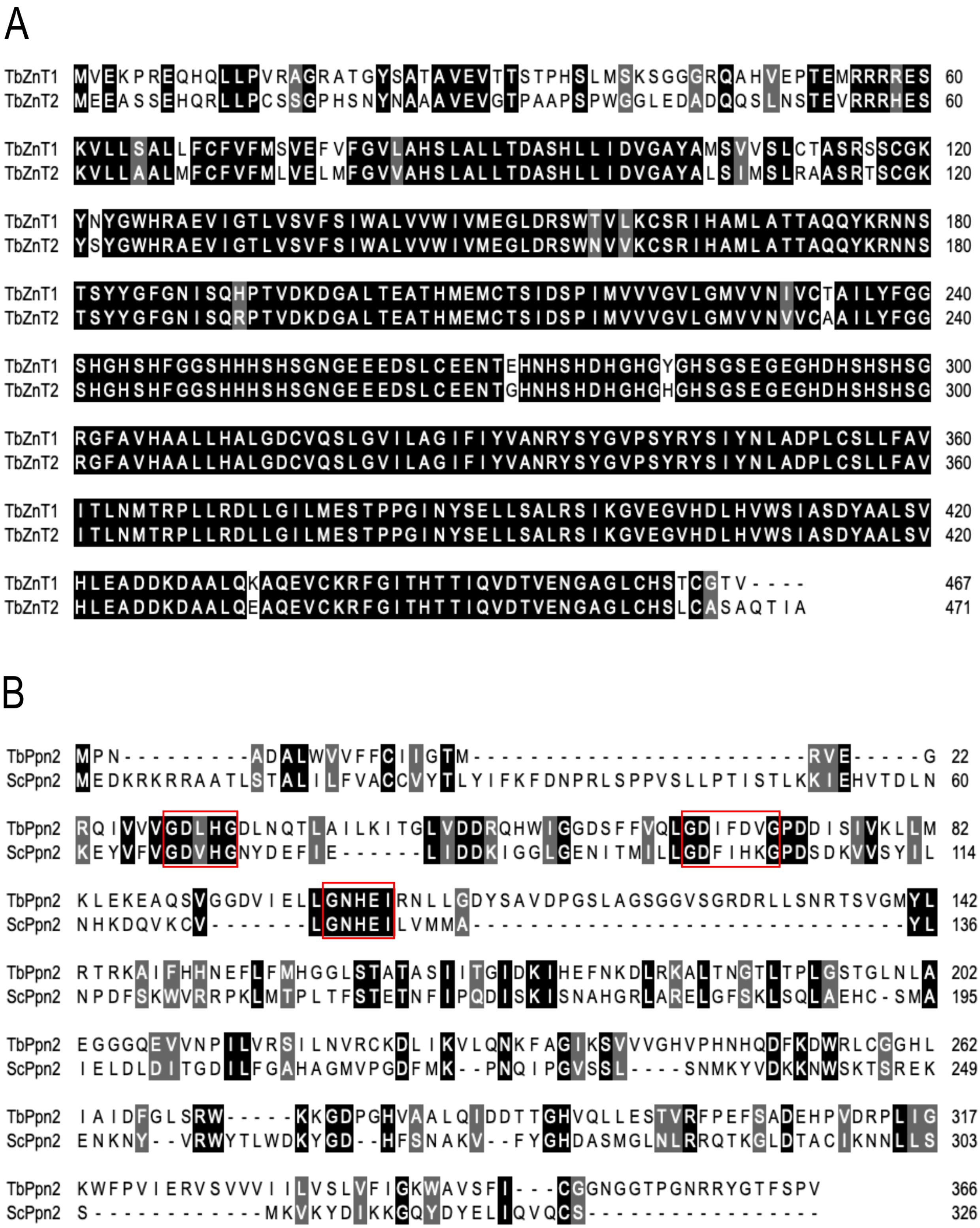

FIG S4 Multiple Sequence Alignment. Sequences of the deduced amino acids of (A) TbZnT1 and TbZnT2 (TriTrypDB: Tb927.4.4960 and Tb927.8.7460), and (B) TbPpn2 (Tb927.6.4630) and its yeast homolog ScPpn2 (GenBank accession no. NP_014182.1) were aligned with MUSCLE

(https://www.ebi.ac.uk/Tools/msa/muscle) and the webserver site (http://www.bioinformatics.org/sms/index.html) (44). Identical (*black*) and similar (*gray*) amino acid residues are shaded. The groups of similar amino acids (ILV, FWY, KRH, DE, GAS, P, C, TNQM) were used for the similarity calculation. The conserved motifs of the catalytic regions of the phosphoprotein phosphatases (PPP)- superfamily are boxed.

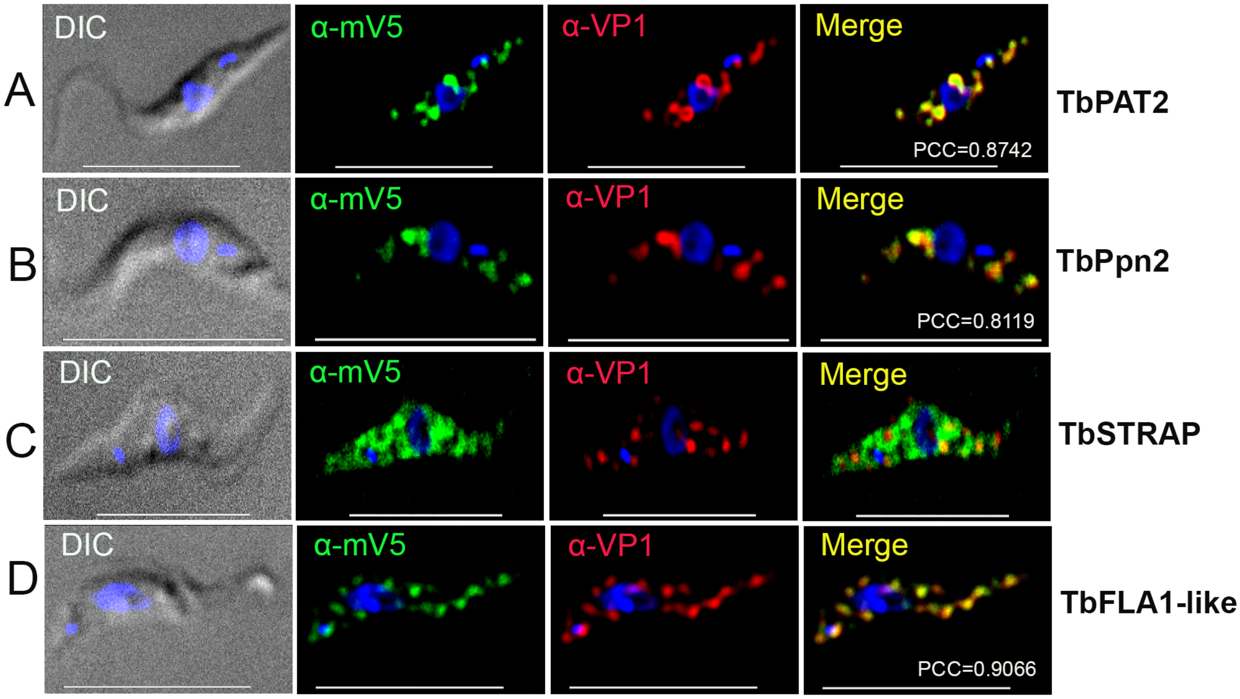

FIG S5 Immunofluorescence microscopy of three enzymes and one membrane protein. TbPAT2 (A), TbPpn2 (B), TbSTRAP (C) and TbFLA1-like (D) were C-terminally smV5-tagged in situ and co-localized with TbVP1 in acidocalcisomes of PCF trypanosomes [Pearson's correlation coefficients (PCC) were 0.8742 (TbPAT2), 0.8112 (TbPpn2), and 0.9066 (TbFLA1-like)]. α-mV5, mouse monoclonal anti-V5 antibody; TbSTRAP has cytoplasmic localization. *Yellow* in merge images indicate co-localization. *Scale bars* for (A–D) = 10 μm.
